# Editorial: The molecular landscape and promising therapeutic targets in aggressive B-cell non-Hodgkin lymphomas

**DOI:** 10.3389/fonc.2023.1278169

**Published:** 2023-08-30

**Authors:** Hanno Maximilian Witte, Axel Künstner, Emil Chteinberg, Francesco Piazza, Gaël Roué, Niklas Gebauer

**Affiliations:** ^1^ Department of Hematology and Oncology, Bundeswehrkrankenhaus Ulm, Ulm, Germany; ^2^ Department of Hematology and Oncology, University Hospital of Schleswig-Holstein, Lübeck, Germany; ^3^ University Cancer Center Schleswig-Holstein, University Hospital of Schleswig-Holstein, Lübeck, Germany; ^4^ Medical Systems Biology Group, University of Lübeck, Lübeck, Germany; ^5^ Institute of Human Genetics Ulm University and Ulm University Medical Center, Ulm, Germany; ^6^ Department of Medicine, Hematology and Clinical Immunology Unit, University of Padova and Myeloma and Lymphoma Pathobiology Laboratory, Veneto Istituto of Molecular Medicine, Padova, Italy; ^7^ Lymphoma Translational Group, Josep Carreras Leukaemia Research Institute, Badalona, Spain

**Keywords:** aggressive B-cell lymphoma, non-Hodgkin lymphoma, genomics, transcriptomics, multi-omics, precision oncology

Precision oncology attempts to translate the results of next-generation sequencing and array-based methods into molecularly stratified therapeutic concepts for cancer. To date, aggressive B-cell non-Hodgkin lymphomas (B-cell-NHL) have received only marginal access to molecularly stratified therapeutic approaches. In addition to the most common entity, diffuse-large B-cell lymphoma (DLBCL), the spectrum of aggressive B-cell-NHL includes 25 other distinct entities. Molecular analyses have been performed in varying degrees for most of these entities, revealing their therapeutic vulnerabilities. However, particularly with the advancements in novel molecular technologies (e.g., single-cell RNA-sequencing, spatial-transcriptomics), some of these entities remain insufficiently characterized. Novel technologies allow a subjacent understanding of molecular tumor-biology, making individual risk-stratification more precise and molecularly stratified therapy recommendations increasingly specific. In addition, new dimensions of tumor-biology, such as phylogenetic evolution, cell-cell interactions or epigenetic regulation of lymphomas can be considered for treatment decision-making in precision oncology.

This editorial presents the results of ten published articles in the *Frontiers in Oncology* Research-Topic “*The Molecular Landscape and Promising Therapeutic Targets in Aggressive B-cell Non-Hodgkin Lymphomas*”.

The original research article by Zhang et al. highlights the prognostic implications of cuproptosis in DLBCL, a copper-induced form of programmed cell-death that is tightly interconnected with mitochondrial metabolism. Here, the authors characterized cuproptosis-related genes (CRG) in DLBCL and identified two biologically and prognostically distinct subtypes. Moreover, the two CRG-clusters differed in terms of immune-cell infiltration and treatment response. The authors constructed a prognostic model incorporating CRG gene-expression and demonstrated the prognostic superiority of this model over the International-Prognostic-Index (IPI).

The success of rituximab in DLBCL treatment is undisputed. To date, the synergistic molecular mechanisms between the CD20-antibody and also those of the next generation (ofatumumab) with components of chemotherapy (CHOP-protocol) are poorly understood. In a preclinical cell-culture approach, Lee et al. addressed this question using transcriptome-sequencing. Based on transcriptional profiles, the authors discovered that CD20-antibodies modulate genes in the JNK and p38 MAPK-family, and thus apoptosis and proliferation of lymphoma-cells through different mechanisms. These findings contribute significantly to our understanding the molecular mechanism of action of CD20-antibodies in DLBCL.

CAR-T-cell therapy is an essential component of novel targeted options for the treatment of lymphoma. The article of the international workgroup of Albendea et al. highlights the existing problems of access and challenges of CAR-T-cell therapy within European healthcare-systems. Comparatively, country-specific measures and solutions are discussed in this manuscript to improve the CAR-T-cell provisioning structure and manufacturing.

In the original research article by Xu et al., panel-sequencing (121 genes) was performed on 259 DLBCL-patients. The molecular alterations were then placed in the context of genomic-clusters according to Wright et al. (1). Subsequently, the presence of dyslipidemia, a risk-factor for cardiovascular disease, was correlated with genomic clusters in DLBCL. Strikingly, DLBCL belonging to the EZB-cluster, in particular, was significantly more frequently associated with dyslipidemia. Pathophysiologically, DLBCL of the EZB-cluster is closely associated with the pathogenesis of follicular lymphoma. Consequently, the rate of dyslipidemia was significantly higher in DLBCL with *BCL2-*fusions (t(14;18)) which represents the genomic hallmark in FL. Ultimately, dyslipidemia had no prognostic impact on overall-survival (OS) in DLBCL.

*NTRK* is an increasingly common therapeutic target in molecular oncology. A separate ESMO-guideline has already been published on this topic. Despite single references indicating that *NTRK* is also detectable in aggressive B-cell-NHL, Ghandili et al. demonstrated on tissue-microarrays that *NTRK* does not represent a promising target for DLBCL-treatment.

At the recent ASH Annual-Meeting, the 5-year update of the REMoDL-B trial was presented, in which DLBCL-patients treated with R-CHOP were randomized to the addition of bortezomib and their benefits were analyzed based on gene expression profiles (whole-genome cDNA-mediated assay) (2, 3). In our Research Topic, Orguirea et al. presents the application of an artificial intelligence approach (LymForest-25 profile) to the data from the REMoDL-B trial, considering not only clinical but also transcriptomic characteristics. The result is a significant improvement in individual risk-stratification, leading to a 30% risk-reduction in half of the (molecularly) high-risk DLBCL-patients in the study.

A subgroup among DLBCL-patients, for whom the greatest challenge in treatment remains to strike an optimal balance between therapy intensity, life-expectancy, and adverse-event management, is represented by geriatric patients. In recent years, the evaluation of nutritional status has become increasingly important with regard to risk stratification of cancer patients. In a comprehensive review article (2,353 cases), Cao and Zhang summarize the available evidence on the Geriatric-Nutritional-Risk-Index (GNRI) and demonstrate that a low GNRI is associated with unfavorable prognosis and disease-progression in elderly and/or frail DLBCL-patients.

Our Research-Topic also included articles on other aggressive B-cell NHL entities. A meta-analysis by Zeremski et al. addressed the still existing question of the value of treatment-intensification in high-grade B-cell lymphomas with MYC and BCL2- and/or BCL6-rearrangements (HGBL-DH/TH). Despite the consideration of some limitations (e.g., retrospective study design, renewed WHO-classification), this meta-analysis provides evidence that chemotherapy-intensification benefits 2-year-PFS and -OS in HGBL-DH/TH.

Plasmablastic lymphoma (PBL) is one of the most aggressive entities in the spectrum of B-cell NHL. Almost no targeted options have been investigated for the treatment of this CD20-negative and rare neoplasm. After progression on first-line chemotherapy, few treatment options are available for these patients representing an urgent unmet clinical need. In a virtual molecular tumor board discussing whole exome and whole transcriptome data from 14 primary-refractory PBL cases, Witte et al. demonstrate that numerous targeted therapeutic vulnerabilities can be evaluated for this difficult-to-treat entity.

The Research-Topic is completed by the presentation of a novel primary renal lymphoma cell line and the corresponding gene expression signature. Li et al. give an outlook on the clinical relevance of this cell line and its application in animal experimental approaches.

The Research-Topic article-collection contains interesting contributions to improve individual risk-stratification, molecular diagnostics and therapeutic implications in aggressive B-cell NHL. On the one hand, the articles demonstrate profound insights into molecular lymphoma biology and on the other hand, they illustrate potential future directions in lymphoma research. The felicitous spectrum of experimental, translational and clinical topics also underlines the multimodality of precision oncology in the field of aggressive B-cell NHL.

## Author contributions

HW: Conceptualization, Data curation, Formal Analysis, Funding acquisition, Investigation, Methodology, Project administration, Resources, Visualization, Writing – original draft, Writing – review & editing. AK: Methodology, Software, Validation, Visualization, Writing – review & editing. EC: Investigation, Resources, Writing – review & editing. FP: Conceptualization, Project administration, Supervision, Writing – review & editing. GR: Conceptualization, Project administration, Supervision, Writing – review & editing. NG: Conceptualization, Data curation, Formal Analysis, Funding acquisition, Investigation, Methodology, Project administration, Resources, Supervision, Writing – review & editing.
